# Gene pool preservation across time and space In Mongolian-speaking Oirats

**DOI:** 10.1038/s41431-024-01588-w

**Published:** 2024-04-11

**Authors:** Natalia Balinova, Georgi Hudjašov, Vasili Pankratov, Erwan Pennarun, Maere Reidla, Ene Metspalu, Valery Batyrov, Irina Khomyakova, Tuuli Reisberg, Jüri Parik, Murat Dzhaubermezov, Elena Aiyzhy, Altana Balinova, Galina El’chinova, Nailya Spitsyna, Elza Khusnutdinova, Mait Metspalu, Kristiina Tambets, Richard Villems, Alena Kushniarevich

**Affiliations:** 1 Research Centre for Medical Genetics, Moskvorechye Str. 1, 115522 Moscow, Russia; 2 Core Facility of Genomics, Institute of Genomics, University of Tartu, Riia 23B, 51010 Tartu, Estonia; 3 Institute of Genomics, University of Tartu, Riia 23B, 51010 Tartu, Estonia; 4 Kalmyk State University named after B. B. Gorodovikov, Pushkina Str. 11, 358000 Elista, Russia; 5 Anuchin Research Institute and Museum of Anthropology, Lomonosov Moscow State University, Mokhovaya Str., 11, 125009 Moscow, Russia; 6 Institute of Biochemistry and Genetics, Ufa Federal Research Center of the Russian Academy of Sciences, 71 Prospekt Oktyabrya Str., 450054 Ufa, Russia; 7 Federal State Educational Institution of Higher Education “Ufa University of Science and Technology”, 32 Zaki Validi Str., 450076 Ufa, Russia; 8 Tuvan State University, Kyzyl, Russian Federation, Lenina Str., 36, 667000 Kyzyl, Republiс of Tuva Russia; 9 Institute of Linguistics, Russian Academy of Sciences, Bolshoi Kislovsky Pereulok, 1, 125009 Moscow, Russia; 10 Institute of Ethnology and Anthropology, Russian Academy of Sciences, Leninsky Prospekt, 32 А, 119334 Moscow, Russia; 11 Estonian Biocentre, Institute of Genomics, University of Tartu, Riia 23B, 51010 Tartu, Estonia

**Keywords:** Population genetics, Haplotypes

## Abstract

The Oirats are a group of Mongolian-speaking peoples residing in Russia, China, and Mongolia, who speak Oirat dialects of the Mongolian language. Migrations of nomadic ethnopolitical formations of the Oirats across the Eurasian Steppe during the Late Middle Ages/early Modern times resulted in a wide geographic spread of Oirat ethnic groups from present-day northwestern China in East Asia to the Lower Volga region in Eastern Europe. In this study, we generate new genome-wide and mitochondrial DNA data for present-day Oirat-speaking populations from Kalmykia in Eastern Europe, Western Mongolia, and the Xinjiang region of China, as well as Issyk-Kul Sart-Kalmaks from Central Asia, and historically related ethnic groups from Altai, Tuva, and Northern Mongolia to study the genetic structure and history of the Oirats. Despite their spatial and temporal separation, small current population census, both the Kalmyks of Eastern Europe and the Oirats of Western Mongolia in East Asia are characterized by strong genetic similarity, high effective population size, and low levels of interpopulation structure. This contrasts the fine genetic structure observed today at a smaller geographic scale in traditionally sedentary populations, and is conditioned by high mobility and marriage practices (traditional strict exogamy) in nomadic groups. Conversely, the genetic profile of the Issyk-Kul Sart-Kalmaks suggests a distinct source(s) of genetic ancestry, along with indications of isolation and genetic drift compared to other Oirats. Our results also show that there was limited gene flow between the ancestors of the Oirats and the Altaians during the late Middle Ages.

**Figure:**
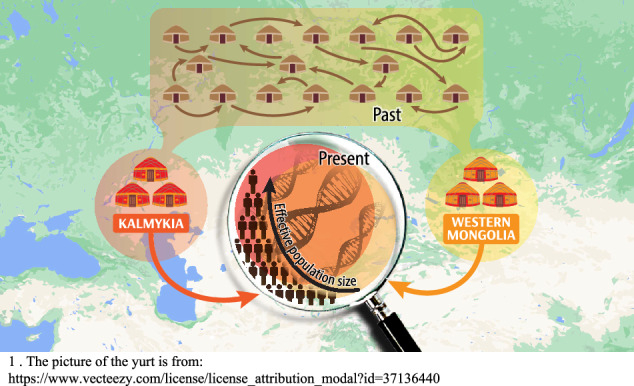
Source of the yurt image: https://www.vecteezy.com/free-vector/yurt.

Source of the yurt image: https://www.vecteezy.com/free-vector/yurt.

## Introduction

The Oirats, a group of closely related peoples who speak the Oirat dialect of the western branch of the Mongolian language, now live far apart on the eastern (western regions of Mongolia, China (XUAR)) and western (Republic of Kalmykia of Russian Federation) edges of the Eurasian Steppe [1, 2]. Both groups include multiple tribes with the largest, Khoshuts, Derbets, and Torguts, present in both groups, and Buzav, which formed in Eastern Europe [3]. Some Kalmak groups in Central Asia, including Sart-Kalmaks in Kyrgyzstan (Sunni Muslims), may also be related to the Oirats through some common traditions and the Oirat dialect of the Mongolian language ([4] but see [5] and [6, 7] for alternative views on the Sart-Kalmak’s origin).

The Oirats are historically related to the mobile nomadic groups in the eastern part of the Eurasian Steppe – today’s Altai region, western Mongolia and northwestern China [1]. The pre-Iron Age genetic history of the region is characterized by ancestral ties to Neolithic individuals of the Devils’ Gate cave in the Far East (ancient northeast Asian ancestry, ANA) [8] as well as connections to Mal’ta and Afontova Gora individuals (ancient north Eurasian ancestry, ANE) [9]. The genetic makeup of the region changed as a result of high population mobility during and after the Iron Age [8]. The Oirats were part of the Great Mongol Empire until the 12th century AD. After its collapse in the 14th-15th centuries AD, some nomadic groups moved westward, forming the Kalmyk ethnic group in the Lower Volga region in the 16th century AD [1]. The uniparental [10–14] and autosomal [15–17] genetic diversity of the Kalmyks brings them close to present-day Central and East Asian populations.

The formation and dispersion of the Oirats is connected with the Altai-Sayan highlands, which were under the influence of several political formations during 13th–18th centuries AD, including the Oirat Khanate [3]. The common history has left traces in the linguistics [18] as well as in the cultural layers of the Oirats and Altaians [3], but their genetic ties have not yet been studied.

The demographic history of the Oirats is poorly understood. In this light, the genomes of modern Oirats are a valuable resource for studying their past. In this study, we generate genome-wide genotypes and mitochondrial DNA sequences of present-day Oirat-speaking groups and historically related South Siberians and analyze them together in the context of modern and ancient human genomes. We aim to characterize the genetic structure of the Oirats, reconstruct their demographic history and genetic relationships with surrounding populations.

## Material

**Fig. 1:**
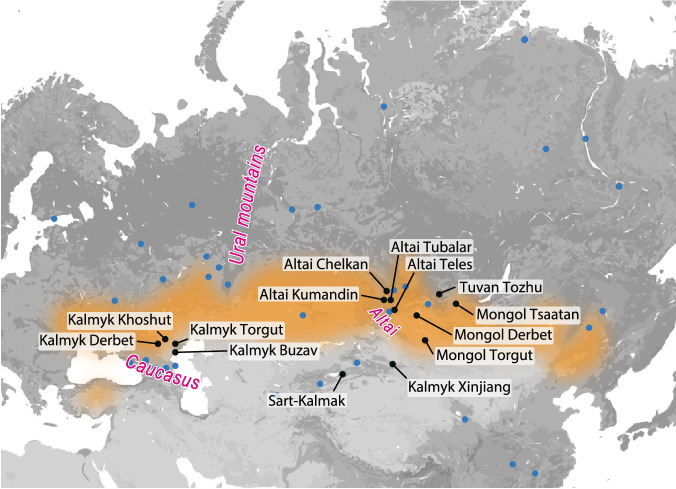
Map showing the geographic origin of the new and reference populations studied for genome-wide diversity. Eurasian Steppe, shaded in orange (schematic representation following https://www.britannica.com/place/the-Steppe). Blue dots indicate the location of the populations from the comparative dataset (Table S1).

Genome-wide genotypes (InfiniumOmniExpress-24v1.2 and v1.3 array) of **80** and mitochondrial DNA (mtDNA) of **453** individuals from different Oirat groups (Kalmyks, Western Mongols), Sart-Kalmaks and South Siberians (Fig.1) were analyzed for the first time in this study (Table S1, Table S2).

## Methods

### Genome-wide data processing and genotype imputation

New samples were pooled with several previously published populations of interest (resulting in *N* = 645) (Table S1). As these were genotyped on different Illumina genotyping platforms, we first performed imputation to increase the cross-platform single nucleotide polymorphism (SNP) overlap. All samples were prepared in a similar way using PLINK v1.9 [19] (SFile). Imputation and phasing were performed separately for each genotyping array on the TOPMed imputation server (https://imputation.biodatacatalyst.nhlbi.nih.gov/). This was followed by post-imputation quality filtering and merging of the datasets with imputed genotypes, resulting in ~915k SNPs in 645 individuals.

### ADMIXTURE and principal component analysis (PCA)

PCA was performed with PLINK v1.9 [19]. The ancestry of each individual was modeled using ADMIXTURE v1.30 [20]. Thirty randomly seeded runs were performed for each number of ancestral populations (*k* = 2–12), and results within each k were post-processed with CLUMPAK to find the consensus solution [21]. Samples belonging to the largest (most frequent) CLUMPAK cluster were grouped by inferred fineSTRUCTURE (FS) populations (see below). For each k, ancestral population proportions within each FS population were averaged and reported. Cross-validation (CV) scores for each k are shown in Fig. S1.

### fineSTRUCTURE, GLOBETROTTER

To investigate genetic clustering between different target groups and other Eurasian populations, we used the ChromoPainter/fineSTRUCTURE (CP/FS) pipeline [22] on our phased and imputed SNP set. FS was run for 3 M Markov chain Monte Carlo (MCMC) iterations (1.5 M burn-in and 1.5 M main iterations) in two parallel runs to assess convergence. The tree-building step was performed as published elsewhere [23] and the run with the highest observed posterior likelihood was used to cluster the samples into genetic groups. The inferred FS groups were further manually inspected and merged into the higher-order FS clusters (Table S1 “FS cluster affiliation”). These FS clusters were used as surrogate populations (Table S1 “GT population name”) to infer admixture proportions and dates with GLOBETROTTER (GT). For additional details on admixture events dating, see SFile and Table S3.

### Fst estimation

We used vcftools-0.1.14 [24] on imputed genomes (460k SNPs, SFile) to obtain Weir and Cockerham’s Fst between each pair of populations across all sites.

### Outgroup *f*3 and *f*4 statistics

Outgroup *f*3 and *f*4 statistics were calculated using the qp3Pop and qpDstat methods, respectively, from ADMIXTOOLS 6.0 program [25]. Ancient DNA (aDNA) samples of interest were extracted from the Allen Ancient DNA Resource [25] (Table S4) and merged with the modern data, resulting in 460k overlapping SNPs (details of aDNA data preparation in SFile). Outgroup *f*3 statistics used in the form *f*3(Mbuti; modern group, ancient group) [26], where modern groups were Kalmyks (Buzav, Khoshut, Torgut), Western Mongols (Derbert, Torgut), Xinjiang Kalmyks, Sart-Kalmak, Buryat, Tsaatan, Tozhu, as well as Altaians (Chelkan, Kumandin, Tubalar, Teles). *f*4 statistics followed the form *f*4(Mbuti, Western Eurasian population; Kalmyk group, Sart-Kalmak). Western Eurasian populations are reported in Table S1.

### Detection of segments identical by descent (IBD)

To detect patterns of long IBD segments sharing between individuals and FS clusters (Table S1), we applied IBIS [27] to 645 imputed genotypes. The choice of IBIS was motivated by the fact that (a) IBIS does not use phase information and is therefore not affected by phase errors, (b) IBIS is to some extent tolerant of genotype errors, and (c) IBIS is applicable to datasets with individuals from different populations. Before running IBIS, we filtered positions based on imputation quality (R2 > = 0.99), resulting in 708 k positions. We ran IBIS with the following arguments:

*-t 10 -maxDist 0.1 -a 0 -mL 5 -mt 300 -er 0.004 -hbd -mLH 5 -erH 0.008*.

This revealed 239,501 IBD segments of 5 centimorgan (cM) or longer. We then used the sum of the genetic length in cM of IBD segments between each pair of individuals as a measure of IBD sharing.

When describing IBD sharing between clusters and/or populations, we removed samples marked “1” in columns “Pruning” in Table S1 (falling into a distinct cluster compared to the majority of samples in that population) and “2” (forming a separate tip within the cluster) from populations and only those marked “2” from clusters.

### Runs of homozygosity

Runs of homozygosity (RoHs) were detected using PLINK v1.9 [19] on the same file as was used for IBD detection (see above). The following arguments were used to obtain the number of RoHs and their total length for each individual: *--homozyg --homozyg-window-snp 100 --homozyg-snp 50 --homozyg-kb 1500 --homozyg-gap 1000 --homozyg-density 50 --homozyg-window-missing 5 --homozyg-window-het 1*.

### Estimating the effective population size (*Ne*) through time

To estimate the effective population size of the Kalmyk and Western Mongol groups, we performed IBDNe [28] on 50 individuals forming the corresponding FS cluster (Table S1). Since the length distribution of IBD segments is fundamental for IBDNe, to avoid segment disruption due to imputation errors, we used only the SNPs that were actually genotyped and not imputed (564k SNPs) for the samples belonging to the cluster that includes the Kalmyks and the Oirats from Western Mongolia. We extracted phase information from our TOPMed imputation results for these positions in these 50 samples. Here, we used the Refined IBD method [28] to detect IBD segments. We then merged segments that were no more than 0.6 cM apart and had no more than one discordant position between two segments to be merged, using the utility provided by the authors of the Refined IBD. Resulting segments longer than or equal to 3 cM were used as input for IBDNe estimation.

### Mitochondrial DNA haplogroup determination

MtDNA haplogroups (hg) were determined by DNA sequencing of the hyper variable segment (HVS) I and HVSII (where necessary) and the screening of 73 coding region markers (Table S2) according to the hierarchy of the mtDNA phylogenetic tree [29] (PhyloTree.org – mtDNA tree Build 17 (18 Feb 2016) http://www.phylotree.org/tree/main.htm). The frequencies of the mtDNA hgs of the studied populations were compared with available comparative data (Table S5) from Eastern Eurasia using Correspondence analysis (CA).

## Results

### Genetic structure of Oirats and South Siberians

**Fig. 2:**
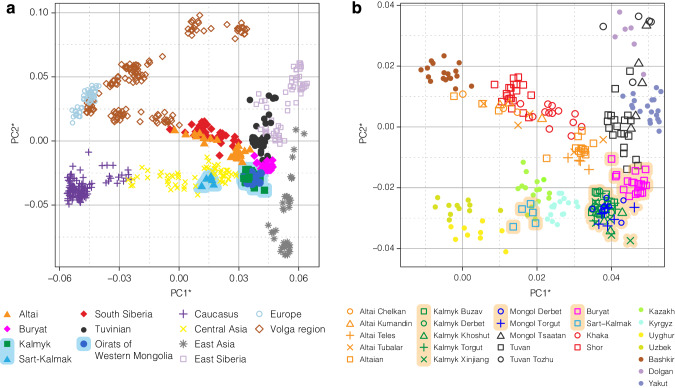
Genetic structure of Oirats and South Siberian populations revealed by PCA. Panel **a** shows a broader Eurasian comparative data set; Panel **b** shows a zoomed-in Eastern Eurasian region with our focus populations. Mongolian-speaking populations are shaded in blue and orange in Panel **a** and **b**, respectively. *Both PC axes were inverted (multiplied by −1) to align with geographical North/South and West/East directions. Details of the reference dataset used are given in Table S1.

To assess the broad genetic profile of the Oirats and South Siberians, we used PCA and Fst. From here on, we will use “Kalmyks” to refer to the Kalmyk groups from Kalmykia and the Xinjiang region of China, and “Oirats of Western Mongolia” (OWM) to refer to the Torgut and Derbet groups from Western Mongolia. Both the PCA results (Fig. 2a, b) and Fst values (Table S6, Fig. S2) show that the Kalmyks are genetically similar to OWM, but are differentiated from their present neighboring populations from Eastern Europe (Fig. 2a, b, Table S6, Fig. S2). The Mongolian-speaking Buryats form their own cluster but are in close proximity to the Kalmyks and OWM. There is no recognizable structure between studied subethnic groups, neither within Kalmykia, nor within the OWM (Fig. 2a, b, Table S6, Fig. S2). The Sart-Kalmaks are distinct from other Oirats: they are grouped together with the Central Asian Kyrgyz, Uyghurs, and Kazakhs (Fig. 2a, b). The Altaians form a cline along the PC1 with northern ethnic groups (Kumandins, Chelkans and Tubalars) shifted towards the West Eurasians, and southern ethnic groups – Teles and Altai-Kizhi – found on the opposite side (Fig. 2a, b). Tsaatans from northern Mongolia lie between Tozhu Tuva and Tuvinians, suggesting varying degrees of admixture between the two groups (Fig. 2a, b).

**Fig. 3:**
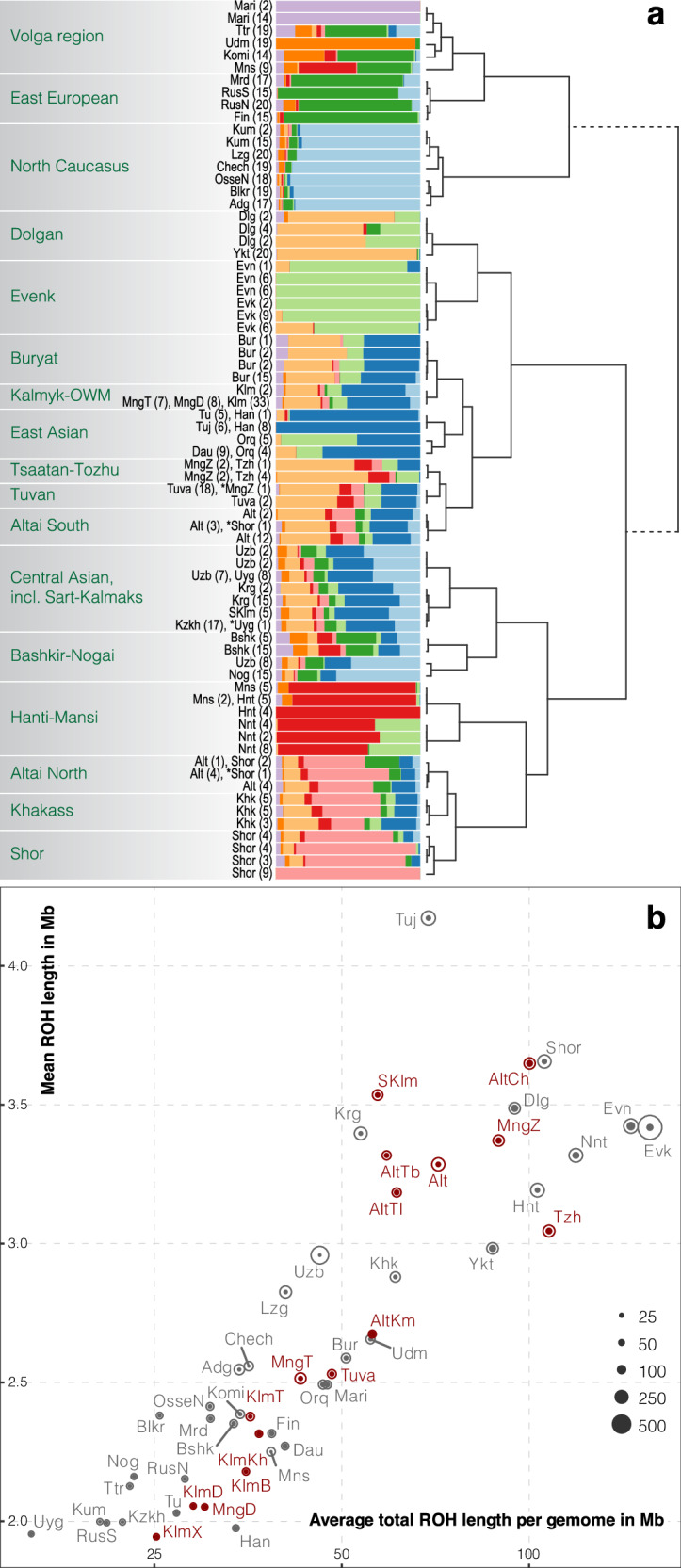
fineSTRUCTURE cluster tree and ancestral components modeled with ADMIXTURE, and total and mean length of runs of homozygosity (RoH) per population. **a** Simplified dendrogram showing the clustering of individuals with similar copying vectors into genetic groups using fineSTRUCTURE. Labels indicate how many samples and which samples are included in each cluster (e.g. “Mari (2)” indicates that the cluster contains two Mari individuals). Barplots show average ancestry proportions in each FS cluster as inferred by ADMIXTURE analysis (*k* = 9). Higher level regional and/or population clusters are shown in dark green. Population abbreviations are: Adg: Adyghe; AltCh: Altai Chelkan; AltKm: Altai Kumandin; AltTl: Altai Teles; AltTb: Altai Tubalar; Alt: Altaian; Blkr: Balkar; Bshk: Bashkir; Bur: Buryat; Chech: Chechen; Dau: Daur; Dlg: Dolgan; Evn: Even; Evk: Evenk; Fin: Finn; Han: Han; KlmB: Kalmyk Buzav; KlmD: Kalmyk Derbet; KlmKh: Kalmyk Khoshut; KlmT: Kalmyk Torgut; KlmX: Kalmyk Xinjiang; Kzkh: Kazakh; Khk: Khaka; Hnt: Khanti; Komi: Komi; Kum: Kumyk; Krg: Kyrgyz; Lzg: Lezgin; Mns: Mansi; Mari: Mari; MngD: Mongol Derbet; MngT: Mongol Torgut; MngZ: Mongol Tsaatan; Mrd: Mordva; Nnt: Nenet; Nog: Nogai; Orq: Oroqen; OsseN: Ossetian; RusN: Russian North; RusS: Russian South; SKlm: Sart-Kalmak; Shor: Shor; Ttr: Tatar; Tu: Tu; Tuj: Tujia; Tuva: Tuvan; Tzh: Tuvan Tozhu; Udm: Udmurt; Uyg: Uyghur; Uzb: Uzbek; Ykt: Yakut (Table S1). **b** Each data point corresponds to the population-average total RoH length per genome (x-axis) and the mean RoH length (y-axis). The areas of the inner filled and outer empty circles are proportional to the minimum and maximum total RoH lengths in each population sample.

At *k* = 9 of the ADMIXTURE analysis, an East Asian component predominates in the Kalmyks and OWM, followed by a Siberian Yakut and Evenk-like component, while West Eurasian ancestry is minor (Fig. 3a, Fig. S1, Fig. S3). The Sart-Kalmaks are closer to Central Asians due to a slight increase in Western Eurasian and a decrease in Siberian Evenk-like components (Fig. 3a). Siberian ancestry (light orange and light green, maximized in Yakuts and Evenks, respectively) predominates among Tozhu from Tuva and Tsaatans from northern Mongolia. The proportions of ancestral components differ between northern and southern Altaian populations: the former have a predominant Shor-like component (pink), whereas the latter have two components - Evenk-like (light green) and Han-like (dark blue) – in almost equal proportions (Fig. 3a). In addition, the Western Eurasian (European-like) component is increased in the Northern Altaic group.

### Patterns of IBD sharing and homozygosity in Oirats and South Siberians

To examine recent gene flow, we analyzed IBD segments shared within and between populations as well as genetic clusters defined by the FS analysis (an individual’s membership in a particular cluster is given in Table S1: e.g. the Kalmyks and OWM are members of the genetic cluster “Kalmyk-OWM”). The values of IBD sharing within populations and within clusters varied widely in our dataset (Fig. S4, Fig. S5). The lowest values within populations and within clusters were detected in most of the East European, Caucasian, Central Asian, and Oirat populations. In particular, the median pairwise IBD sharing is 25 cM within the Kalmyk-OWM cluster, while extended IBD runs were observed in the South Siberian populations, suggesting low *Ne* (e.g., the median IBD sharing reaches 200 cM and higher within the Tuvan-Tozhu and Altai North clusters), which is expected for second cousins who share a couple of ancestors from around three generations ago [30] (Fig. S4, Fig. S5).

**Fig. 4:**
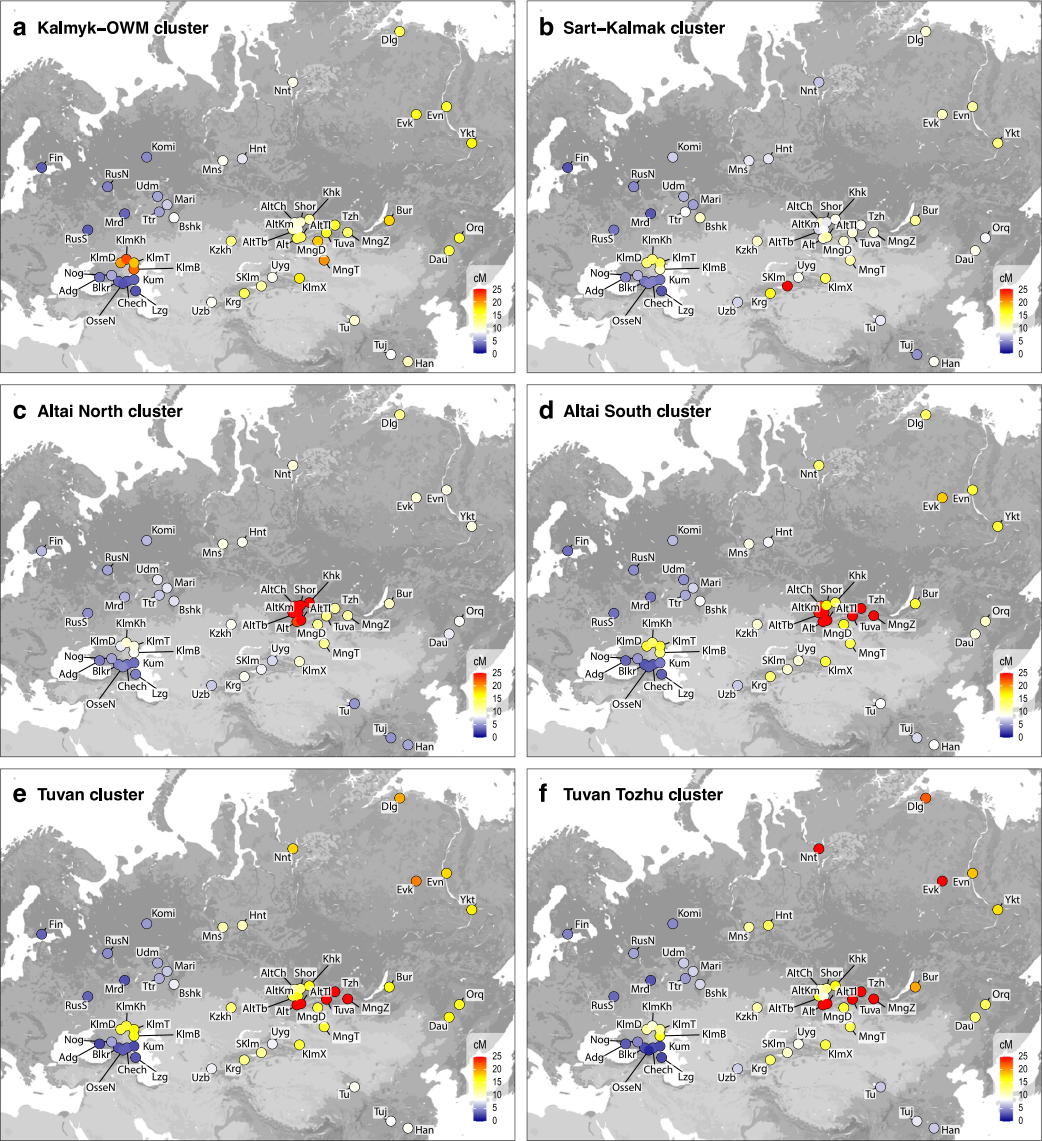
Population-median IBD sharing with fineSTRUCTURE-defined clusters. The color of each dot indicates the distribution of IBD between the corresponding population (Table S1 explains population abbreviations) and the following FS clusters: **a** Kalmyk-OWM, **b** Sart-Kalmak, **c** Altai North, **d** Altai South, **e** Tuvan, **f** Tuvan-Tozhu. The color scale in each panel is capped at 25 cM to improve resolution at the lower end. To obtain the values to be plotted, we first calculated the average sharing with the cluster members individually for each sample from a given population and then took the median of the values in each population (this corresponds to the median shown in the boxplots). This is motivated by the fact that population groups in general are more heterogeneous than clusters.

Individuals forming the Kalmyk-OWM cluster have the highest total IBD length, with East Asians, Central Asians, and South and East Siberians, with Buryats standing out among other Siberian groups. This is similar for Sart-Kalmaks, but the total length of IBD segments is highest with Kyrgyz (Fig. 4, Fig. S6, Fig. S7). IBD-relatedness is different for the Altai North and Altai South clusters: while the former is more localized, the latter shares more IBD with a wide range of Siberian, Central Asian populations and Kalmyks (Fig. 4, Fig. S6, Fig. S7). The Tuvan-Tozhu have more IBD in common with the Siberian populations – the Buryats, Dolgans, Evenks, and Nenets – than with the Tuvans (Fig. 4, Fig. S6, Fig. S7).

We have examined the total length of RoH, as a measure of a homozygosity in our dataset (Fig. 3b). Both the Kalmyks and OWM have comparatively low values of total RoH (Fig. 3b). Among the Kalmyk subethnic groups, the lowest RoH values were found among Derbets and Kalmyks from Xinjiang, and the highest values were found among Khoshuts. Notably, the total RoH length in Tsaatans from northern Mongolia, unlike other Mongolian populations studied, is among the highest in our dataset. RoHs in this population are also longer on average than in other populations with similar total RoH length, suggesting low *Ne* in the relatively recent past.

In contrast to the Kalmyks and OWM, Sart-Kalmaks from Central Asia have both higher total and mean RoH lengths, which may reflect a more recent decrease in population size during the westward migration of some of their ancestors into Kyrgyz territory [5]. Higher levels of homozygosity are also a characteristic of both northern and southern Altaic populations, among which Altaic Chelkans stand out (Fig. 3b). Finally, the Tozhu people of the Republic of Tuva have the highest values of total RoH length among the new populations generated in this study, indicating a small population size of these groups, probably due to geographical isolation or bottleneck (Fig. 3b). In the case of Tozhu, however, the high total RoH length is due to a large number of relatively short segments, suggesting a recent increase in *Ne* or exogamy [31].

### Effective population size dynamics for the Kalmyk-OWM cluster

Modeling of *Ne* in the Kalmyk-OWM cluster suggests its rapid increase starting about 20 generations ago (if generation = 30 years, then about 600 years ago) (Fig. S8) [27]. However, these results should be treated with caution because (a) the number of samples used is relatively small, and (b) we had to combine the Kalmyk and OWM to achieve a sample size of at least 50 individuals, which may have inflated *Ne* estimates in the very recent past. Nevertheless, the recent population expansion is consistent with the low levels of IBD sharing observed in the Kalmyk-OWM cluster, as well as in individual Kalmyk and OWM populations.

### Exploring admixture events in Oirats and South Siberians

Admixture events in Kalmyks, OWM, Sart-Kalmaks, and South Siberian genetic clusters were modeled using GT and MALDER analyses. Simple one-date admixture events between two source populations dated to the 13th–14th centuries AD are inferred by GT with medium to high certainty (goodness-of-fit R2 > 0.6) in most of the clusters, but Mongolian Tsaatans (Table S7a, b). MALDER results further confirm these findings: with the exception of three groups (Altai South, Tuvan-Tozhu, and Kalmyk Khoshut), it detected an admixture event between the eastern and western Eurasian reference groups. This pattern and admixture dates overlap with those in the GT analysis (see SFile for potential explanation of the observed differences in dates).

The Kalmyk, OWM, and Sart-Kalmak groups could be represented as a mixture of East Eurasian (Daur/Oroqen/Buryat proxies) (65–80%) and West Eurasian (South Russian/Caucasian proxies) sources (20–35%) (Table S8). The proportion of West Eurasian ancestry among Sart-Kalmaks is significantly higher than among Kalmyks and OWM (35%), while Buryat-like ancestry is lower (10%) (Table S8, Table S9). At the level of ethnic subgroups of Kalmyks, a slight variation in West Eurasian ancestry is observed (Table S8). Admixture between Central Asian (Uzbeks/Kazakhs) and South Siberian (Tuvan/Shor) proxies was modeled in the two Altai clusters (Table S8).

### Relatedness to ancient human groups

Analysis of the outgroup *f*3 statistics shows that Kalmyks, OWM, Sart-Kalmaks, Buryats, and Tsaatans from northern Mongolia share a high number of derived alleles with ancient groups whose ancestry is classified as ANA (Ancestral Northeast Asian) (Fig. S10, Table S4, Table S10). These include: (a) pre-Bronze Age (BA) groups from central-eastern Mongolia, Priamurie (Devil’s Gate), Buryatia (Fofonovo site), and the Baikal region (Lokomotiv Early Neolithic site, Shamanka Early BA, Ust Belaya Early BA), in which ANA predominates; (b) Middle-Late BA (MLBA) and Early Iron Age groups from central and eastern Mongolia (Ulaanzuukh site; Slab Grave culture), the Baikal region (mixture of ANA and ANE (Ancestral North Eurasian) ancestry related to Mal’ta/Afontova Gora individuals); (c) Late Xiongnu groups and late medieval individuals (Khitan, Mongol) from Mongolian territory.

Present-day Altaians and Tuva Tozhu are closer to those ancient groups that represent a mixture of ANE and ANA genetic ancestries (Fig. S10, Table S10). These include primarily Neolithic and Early BA individuals from the Baikal region, but also those where ANA is predominant (pre-BA, MLBA, and Early IA individuals from Mongolian territory). Chelkans also share a higher number of derived alleles with West Siberian hunter-gatherers (HG) (Sosnovyj Ostrov, Tumen) and from Karelia (Fig. S10, Table S10).

### Mitochondrial DNA diversity in Oirats and South Siberians

We characterized the mtDNA diversity of nine Oirat ethnic groups - four groups of Kalmyks from Kalmykia, three groups from western Mongolia, Kalmyks from the Xinjiang region of China, and Sart-Kalmaks from Kyrgyzstan - together with Mongolian Tsaatans and Tozhu Tuvans, who speak the Tozhu dialect of Tuvan (Table S2). All Kalmyk subethnic groups, as well as OWM and Tsaatans from Mongolia, are characterized by quite diverse maternal gene pools. In contrast, Tozhu Tuvans have a limited number of hgs, typical of a founder event followed by isolation and small population size (Fig. S9a).

East Eurasian hgs (B, F, R9b, A, Y, N9a, C, Z, D, G, M7, M9a, M13) form the largest common component (more than 70%) among all ethnic groups studied (Fig. S9a). The frequency and composition of these hgs differ among the groups. Hg D is the most common among both Kalmyks and OWM. Hg G occurs more frequently in Mongol Khoshuts, Mongol Derbets, Sart-Kalmaks, and Xinjiang Kalmyks than in the other groups studied. Mongol Tsaatans and Tozhu Tuvans differ from other populations by high frequency (> 50%) of hg C and low frequency of hg D.

West Eurasian hgs (HV, R1, R2, JT, U, N1, N2, X) form the minor component in the groups studied, while they are virtually absent in the Tozhu Tuvan and Mongol Khoshut. Western Eurasian hgs constitute > 20% of the maternal gene pool of the Kalmyk Buzav and Torgut, and Sart-Kalmak, but only 8% among Mongol Torguts, Mongol Tsaatans and Xinjiang Kalmyks. However, considering the small sample sizes of some sub-groups, the differences should be taken with caution. In the Eurasian background, the Oirats studied are intermingled with South Siberians and Central Asians and are clearly separated from modern Caucasian populations (Fig. S9b).

## Discussion

### Genetic ancestry of Oirats across the Eurasian Steppe

The ancestors of the modern Kalmyks and the OWM lived in the region that is now western Mongolia, northwestern China, eastern Kazakhstan, and southern Siberia until the 16th century AD, when a number of tribes began to migrate westward, reaching the lowlands of the Volga and Don rivers in eastern Europe in the 17th century AD [32–36].

The high genetic similarity of Kalmyks and OWM (Fig. 2, Fig. 3a, Table S6, Fig. S7, Table S8) supports a common genetic ancestry for these groups and its high degree of preservation over time and distance. Some factors that would support the preservation of genetic ancestry in Kalmyk ancestors are: (1) high mobility of pastoral nomads from the Eurasian Steppe of the 16th–17th centuries AD – within about 50 years they reached the lower Volga and Don rivers from Dzungaria [3, 37, 38], (2) pastoral nomadic lifestyle maintained until the 20th century in the regions of the lower Volga and Don rivers [3, 37, 38], (3) language (Oirat dialect of the Mongolian language) [3, 37, 39], and (4) religion (Lamaist Buddhists) [3, 37, 39] could limit gene flow with encountered populations with different traditions.

Oirat groups (Kalmyks and OWM), as well as Sart-Kalmaks and Buryats, are closely related to ancient groups that inhabited regions east of the historical land of the Oirats (Fig. S10). This suggests a deep common ancestry among these groups, consistent with the proposed importance of the eastern Transbaikal and Amur regions in the formation of proto-Mongolian tribes [40]. Similarly, genetic links between present-day Oirats and Altaians go back as far as the Neolithic and Bronze Ages, suggesting a common prehistoric ancestry between these populations (Fig. S10). There is little evidence of substantial gene flow between the Oirat and Altai ancestors during the late medieval/early modern period, despite the Oirat political dominance in the region at the time.

The Kalmyks and OWM are likely to have originated from a parental population(s) that has maintained a relatively large effective population size (*Ne*) throughout its history (Fig. 3b) (also observed earlier on uniparental data by Nasidze et al., 2005) [14]. The *Ne* curve estimated in the joint cluster that includes Kalmyks and OWM (Fig. S8) suggests a population growth around 600 y.a. This estimate overlaps with the period of expansion of the Mongol Empire, when numerous tribes became a part of this state in the 12th––13th centuries AD [3, 37, 38]. Importantly, IBDNe estimations reflect the population growth itself, rather than a mixture of different populations [41]. This, together with the genetic evidence in our study, may suggest that the expansion of the Mongols and the Oirats in particular, involved not only the absorption of a variety of different populations, but also actual population growth.

The gene pool of the Sart-Kalmaks demonstrates high similarity with Central Asians, signs of founder effect and isolation of their ancestral population for some period of time (Fig. 2a, b, Fig. 3a, b, Fig. S5, Fig. S7, Table S6, Table S9). Distinct genetic sources for Oirats and Issyk-Kul Sart-Kalmaks, and/or intensive admixture would be consistent with the observed genetic differences between the two groups. However, reconstructing the history of the Sart-Kalmaks will require a more comprehensive dataset and modeling of the population genetic history.

### Social traditions strongly influence the genetic structure of (ex)nomadic Oirats

Previous studies have demonstrated the presence of genetic structure mirroring the geographic origin of individuals in populations with historically agricultural economies (e.g. Estonians, Poles) [41, 42]. The pattern we observe for the studied Oirats, whose recent ancestors were pastoral nomads, is the opposite: Kalmyk and Western Mongolian groups are genetically very similar to each other, despite the large geographic distance that now separates them (Fig. 2, Fig. 3, Fig. S7, Table S6). Moreover, we reveal little to no differences between tribal groups, both within Kalmyks and OWM (Fig. 2a, b, Fig. 3a, Table S2) [13]. The minor differences we observe between the groups (Caucasian component in the Kalmyk Torguts, slight increase in West Eurasian ancestry in the Buzav (Table S8)) are less likely to be due to genetic drift (low within-group IBD sharing as well as growth in *Ne* during the last 600 years (Fig. 4, Fig. S8)), but can be explained by limited gene flow from neighboring populations.

The lack of differentiation between Kalmyks and OWM indicates that they have maintained large *Ne* since their divergence. In addition to recent population growth, exogamy may also contributed to preventing isolation between local ethnic subgroups. Exogamy (“marriage outside the kin group”) is an ancient custom dating back to proto-Mongolian tribes, that served to prevent marriages between related people [37, 43]. Although modified over time (reckoning the number of generations to common ancestor; geographic distance, clan affiliation etc), exogamy has been preserved to the present day and is common among numerous populations living throughout the Great Eurasian Steppe (Mongolians, Oirats, Tuvans, Kazakhs, Kyrgyz, Bashkirs) [37, 43]. Although we have not tested in our study the hypothesis of exogamy explicitly, the genetic patterns we observe are compatible with it - the lower total length and the lower number of RoHs in the present-day Kalmyks (Fig. 3b).

In summary, the present-day Oirats of Western Mongolia and Kalmykia represent a genetic unity that traces its deep ancestry to eastern Transbaikal and the Amur River region. The revealed low intrapopulation structure and relatively high effective population size is consistent with the tradition of exogamy among historically nomadic pastoralists. In contrast, the genetic profile of the Issyk-Kul Sart-Kalmaks may suggest different source(s) of genetic ancestry(ies), as well as indications of isolation and genetic drift, compared to other Oirats. Our study does not provide evidence of significant gene flow between Oirat ancestors and populations from the Altai-Sayan highlands of southern Siberia during the late Middle Ages.

### Supplementary information

SFile

Fig.S1

Fig.S2

Fig.S3

Fig.S4

Fig.S5

Fig.S6

Fig.S7

Fig.S8

Fig.S9

Fig.S10 A-O

TableS1.

TableS2.

TableS3.

TableS4.

TableS5.

TableS6.

TableS7ab.

TableS8.

TableS9.

TableS10.

## Data Availability

The newly generated genome-wide genotype data generated in this study are available at https://www.ncbi.nlm.nih.gov/geo/(accession numbers GSE262748 and GSE262754) and the data depository of the Estonian Biocentre (https://evolbio.ut.ee/).
