# Supplementary material for: Gene pool preservation across time and space In Mongolian-speaking Oirats
Source: Eur J Hum Genet. 2024 Apr 11;32(9):1150–8. doi: 10.1038/s41431-024-01588-w (PMC11369229; doi:10.1038/s41431-024-01588-w)
Supplement: Supplementary file 1 — SFile [file 41431_2024_1588_MOESM1_ESM.docx]

**Gene Pool Preservation Across Time And Space In Mongolian-speaking Oirats**

Supplementary Data

**Material and Methods**

DNA was isolated from peripheral blood leukocytes using the standard phenol-chloroform method [(Mathew, 1984)](https://paperpile.com/c/ESP5nu/yIVk). All procedures performed in the study involving human participants were in accordance with the ethical standards of the institutional and/or national research committee and with the 1964 Helsinki declaration and its later amendments or comparable ethical standards. Informed consent was obtained from all individual participants included in the study. This genetic and epidemiological study was approved by the Internal Review Board of the Research Centre for Medical Genetics (ProtocolNo. 5/2 dated February 9, 2015).

**Genome-wide data pre-processing and genotype imputation**

*Datasets preprocessing*

Samples within each genotyping array were merged and processed together. Prior to merging, indels and A/T and G/C variants were removed to avoid potential strand bias. Within each genotyping array batch, samples with more than 5% of missing data were removed, and only common variable autosomal single nucleotide polymorphisms (SNPs) (minor allele frequency (MAF) > 1%) with less than 5% of missing data were kept for further analysis. Each individual batch was checked for strand, allele, position and GRCh37 REF/ALT allele assignment problems using “HRC or 1000G Imputation preparation and checking” pipeline v4.3.0 (https://www.well.ox.ac.uk/~wrayner/tools/) and HRC reference panel v1.1 (HRC.r1-1.GRCh37.wgs.mac5.sites.tab, <ftp://ngs.sanger.ac.uk/production/hrc/HRC.r1/>).

*Imputation*

The number of input SNPs for the imputation process varied between 483k and 739k for the five different genotyping arrays. The imputation and phasing was performed on the TOPMed imputation server for each genotyping array separately (<https://imputation.biodatacatalyst.nhlbi.nih.gov/>). After the imputation, only SNPs with imputation quality score R2>0.98 were kept for each batch. Next, five imputed genotyping batches were merged together and only common (MAF>0.01) bi-allelic SNPs overlapping between all five batches were kept. Approximately 915k SNPs passed this post-imputation filter. This filtering was performed with BCFtools v1.9 [(Danecek et al. 2021)](https://paperpile.com/c/ESP5nu/3K4M).

*Ancient DNA data preparation*

Ancient DNA genotype data extracted from from the Allen Ancient DNA Resource [(Mallick et al. 2023)](https://paperpile.com/c/ESP5nu/JA5w) were converted to vcf format with PLINK (*--recode –a2-allele 4 3 ‘#’*), A/T and C/G transversions were removed prior to correcting the REF/ALT alleles with BCFtools v1.9 [(Danecek et al. 2021)](https://paperpile.com/c/ESP5nu/3K4M). Liftover from hg19 to hg38 was done using Picard (<http://broadinstitute.github.io/picard>). The vcf file was converted back to bed and then merged with the modern data set, keeping only variants present in both ancient and modern data (460,418 in total).

*Filters applied on dataset before Principal Component Analysis (PCA) and ADMIXTURE*

Before ADMIXTURE and PCA analyses, additional filtering was performed by removing SNPs in high linkage disequilibrium (R2>0.4 in sliding window of size 100 SNPs, window step size = 10 SNPs, *plink --indep-pairwise 100 10 0.4*) using PLINK (Purcell et al., 2007).

*Filters applied on dataset before FST estimation*

Before FST estimation, following filters were applied to the imputed dataset: 1) imputation quality (R2>=0.99); 2) removing SNPs with R2>0.4 in a sliding windows of 10,000 SNPs with a step of 1,000 SNPs (*--indep-pairwise 10000 1000 0.4*) using PLINK (Purcell et al., 2007).

**Inferring admixture proportions and dating admixture events**

Globetrotter (GT) [(](https://paperpile.com/c/ESP5nu/KV6Tb)[Hellenthal](http://paperpile.com/b/ESP5nu/KV6Tb) et al., 2014[)](https://paperpile.com/c/ESP5nu/KV6Tb) was used to detect signals and dates of admixture for the target populations groups defined using the approach described above. This framework does not require the specification of source populations of admixture, but instead models these sources from a given set of modern sampled surrogate populations (TableS1). GT inference was performed using a “regional” approach, where each target population was mapped, painted and analyzed using a unique set of donor populations. Neighboring populations with ancestry closely shared with the target populations groups were excluded from potential admixture sources to reveal ancient and subtle admixture patterns in the target populations (TableS3).

To confirm the findings of the GT analysis, we utilized a different admixture detection method called MALDER (Pickrell J. 2015. MALDER. Available from: <https://github.com/> joepickrell/malder), which is an extension of ALDER (Loh et al., 2013). We conducted tests for admixture in each target group using all possible pairwise combinations of reference populations. We report statistically significant results of admixture using the MALDER curve with the greatest amplitude (Table S7ab).

The difference between the exact sources of admixture as detected by MALDER and GT can be explained by the fact that the haplotype-based GT framework does not require the specification of source populations of admixture, but instead reconstructs them from the given set of sampled surrogates (TableS1), while LD-based MALDER uses modern reference populations as direct proxies of ancient admixture events. Detection of complex multi-date or multi-way admixture events remains challenging (<https://academic.oup.com/bib/article/21/1/144/5168590>) which can explain discrepancies between GT and MALDER results.

**References**

[Mathew CGP. The Isolation of High Molecular Weight Eukaryotic DNA. *Nucleic Acids*. 1984;31–4.](http://paperpile.com/b/ESP5nu/yIVk)

[Danecek, Petr, Adam Auton, Goncalo Abecasis, Cornelis A. Albers, Eric Banks, Mark A. DePristo, Robert E. Handsaker, et al. 2011. “The Variant Call Format and VCFtools.” *Bioinformatics* 27 (15): 2156–58.](http://paperpile.com/b/ESP5nu/5myR)

[Mallick, Swapan, Adam Micco, Matthew Mah, Harald Ringbauer, Iosif Lazaridis, Iñigo Olalde, Nick Patterson, and David Reich. 2023. “The Allen Ancient DNA Resource (AADR): A Curated Compendium of Ancient Human Genomes.” *bioRxiv : The Preprint Server for Biology*, April. https://doi.org/](http://paperpile.com/b/ESP5nu/JA5w)[10.1101/2023.04.06.535797](http://dx.doi.org/10.1101/2023.04.06.535797)[.](http://paperpile.com/b/ESP5nu/JA5w)

[Purcell, Shaun, Benjamin Neale, Kathe Todd-Brown, Lori Thomas, Manuel A. R. Ferreira, David Bender, Julian Maller, et al. 2007. “PLINK: A Tool Set for Whole-Genome Association and Population-Based Linkage Analyses.” *American Journal of Human Genetics* 81 (3): 559–75.](http://paperpile.com/b/ESP5nu/l79S)

[Hellenthal G, Busby GBJ, Band G, Wilson JF, Capelli C, Falush D, et al. A genetic atlas of human admixture history. *Science*. 2014 Feb 14;343(6172):747–51.](http://paperpile.com/b/ESP5nu/KV6Tb)

Loh P-R, Lipson M, Patterson N, Moorjani P, Pickrell JK, Reich D, and Berger B. [Inferring Admixture Histories of Human Populations Using Linkage Disequilibrium.](http://www.genetics.org/content/193/4/1233.full) *Genetics,* 2013.

**Supplementary figures with full legends**

**Fig.S1** **CV scores for each k of the ADMIXTURE analysis.**

**Fig.S2 FST values between populations calculated from genome-wide data.** TableS1 explains population abbreviations.

**Fig.S3** **Individual-wise ADMIXTURE plot for k3-12**. TableS1 explains population abbreviations.

**Fig.S4** **Intra-cluster IBD-sharing.**

Each data point corresponds to IBD sharing (total length of IBD segments) between a pair of individuals within a cluster. The box itself shows the 25th, 50th and 75th percentiles while the whiskers correspond to the lowest and highest values within 1.5xIQR (IQR – interquartile range) and the remaining data points are shown as individual points.

**Fig.S5** **Intra-population IBD-sharing.**

Each data point corresponds to IBD sharing (total length of IBD segments) between a pair of individuals within a population. The box itself shows the 25th, 50th, and 75th percentiles, while the whiskers correspond to the lowest and highest values within 1.5xIQR (IQR – interquartile range) and the remaining data points are shown as individual points. TableS1 explains population abbreviations.

**Fig.S6** **IBD-sharing between populations and fineSTRUCTURE-defined clusters.** Each data point corresponds to the average sharing of an individual from a given population with all the individuals in the corresponding cluster: a) Kalmyk-OWM, b) Sart-Kalmak, c) Altai-North, d) Altai-South, e) Tuvan, f) Tuvan-Tozhu. The box itself shows the 25th, 50th, and 75th percentiles, while the whiskers correspond to the lowest and highest values within 1.5xIQR (IQR – interquartile range) and the remaining data points are shown as individual points. TableS1 explains population abbreviations.

**Fig.S7** **Per pair IBD-sharing.**

Total IBD sharing (sum of all shared segments longer than 5 cM) between each pair of individuals belonging to clusters a) Kalmyk-OWM, b) Sart-Kalmak, c) Altai-North, d) Altai-South, e) Tuvan, f) Tuvan-Tozhu, as defined by the fineSTRUCTURE analysis. TableS1 explains population abbreviations.

**Fig.S8 Changes in the effective population size (*Ne*) of the meta-population corresponding to the Kalmyk-OWM cluster as defined by fineSTRUCTURE.**

The curve shows *Ne* estimates per generation as revealed by IBDNe. The shaded area shows 95% confidence intervals.

**Fig.S9** **Distribution of mtDNA haplogroups in Oirats and South Siberians (A) and their position in the Eurasian genetic background (B).** The frequencies of mtDNA haplogroups in Eurasian populations are listed in TableS5. Population abbreviations are: Chamalals (Cha), Chechens (Che), Dargins (Drg), Ingush (Ing), Kumyks (Kum), Lezgins (Lez), Tabasarans (Tab), Kuban Nogays (Nog), Kara Nogays (Knog), Abazins (Ab), Adyghe (Ad), Balkars (Bal), Cherkessians (Chr), Kabardin (Kab), Karachays (Kar), North Ossetians (NO), Abkhazians (Abk), Armenians (Arm), Georgians (Go), South Ossetians (SO), Kalmyk Buzav (KBV), Kalmyk Derbet (KDR), Kalmyk Khoshut (KK), Kalmyk Torgut (KT), Mongol Derbet (MDR), Mongol Khoshut (MK), Mongol Torgut (MT), Mongol Tsaatan (Zaa), Sart Kalmak (SK), Tozhu Tuvan (ToT), Xinjiang Kalmyk (XK).

**Fig.S10** **A-O** **Outgroup *f*3 statistics with ancient human groups.**
