# Supplementary figures and images for: Gene pool preservation across time and space In Mongolian-speaking Oirats
Source: Eur J Hum Genet. 2024 Apr 11;32(9):1150–8. doi: 10.1038/s41431-024-01588-w (PMC11369229; doi:10.1038/s41431-024-01588-w)

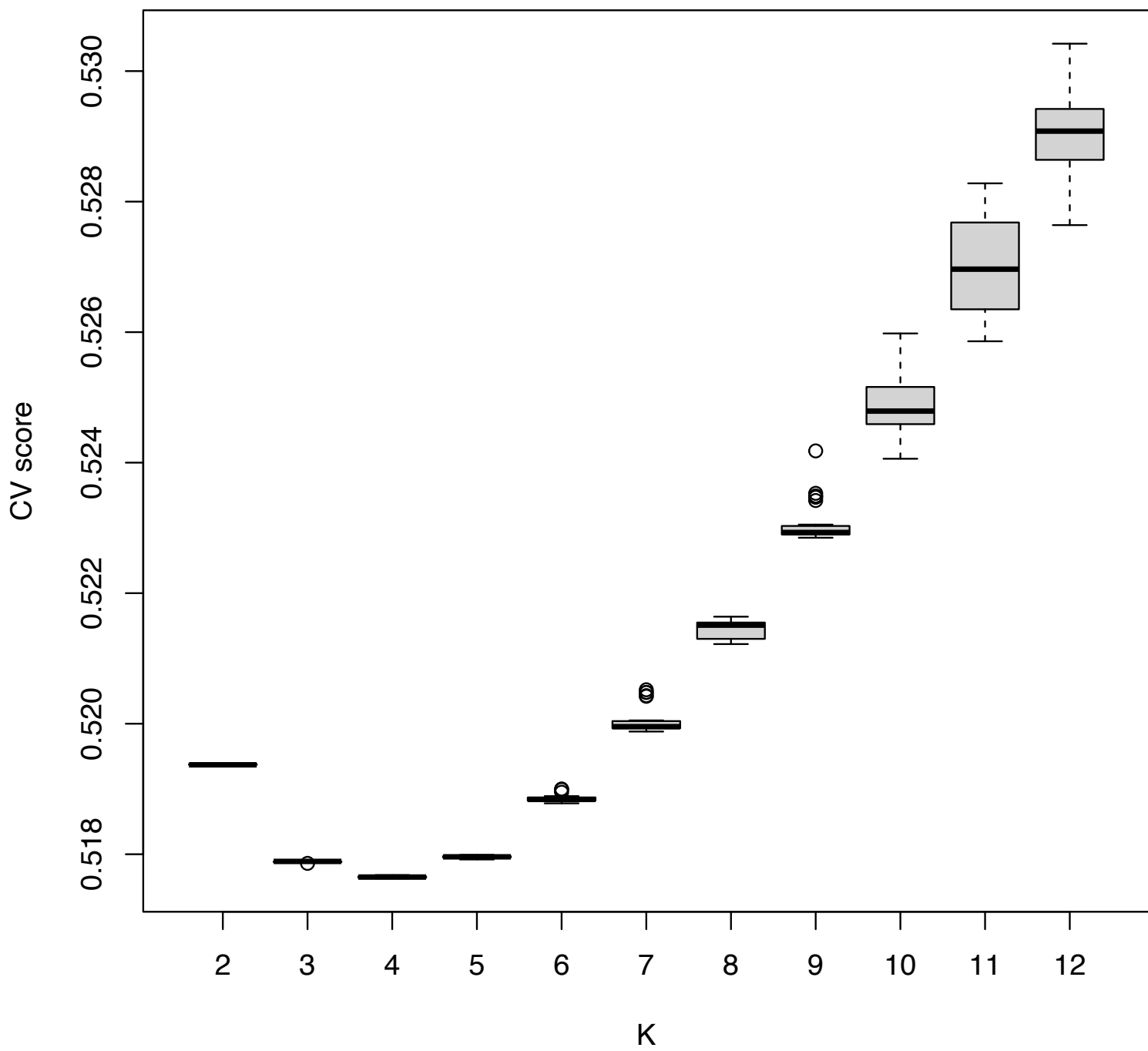

Supplement: Supplementary file 2 — Fig.S1 [file 41431_2024_1588_MOESM2_ESM.pdf]

Fst between pops

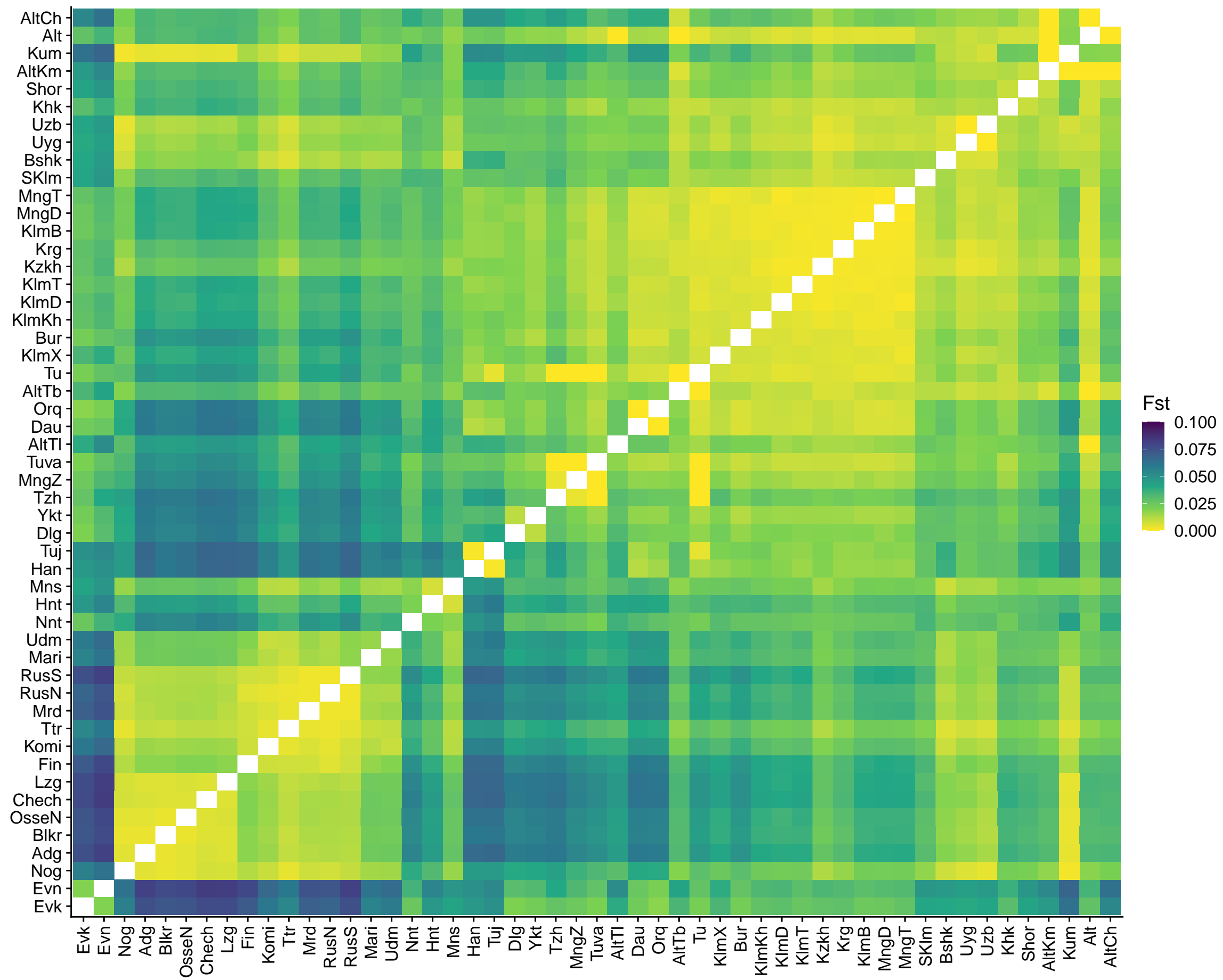

Supplement: Supplementary file 3 — Fig.S2 [file 41431_2024_1588_MOESM3_ESM.pdf]

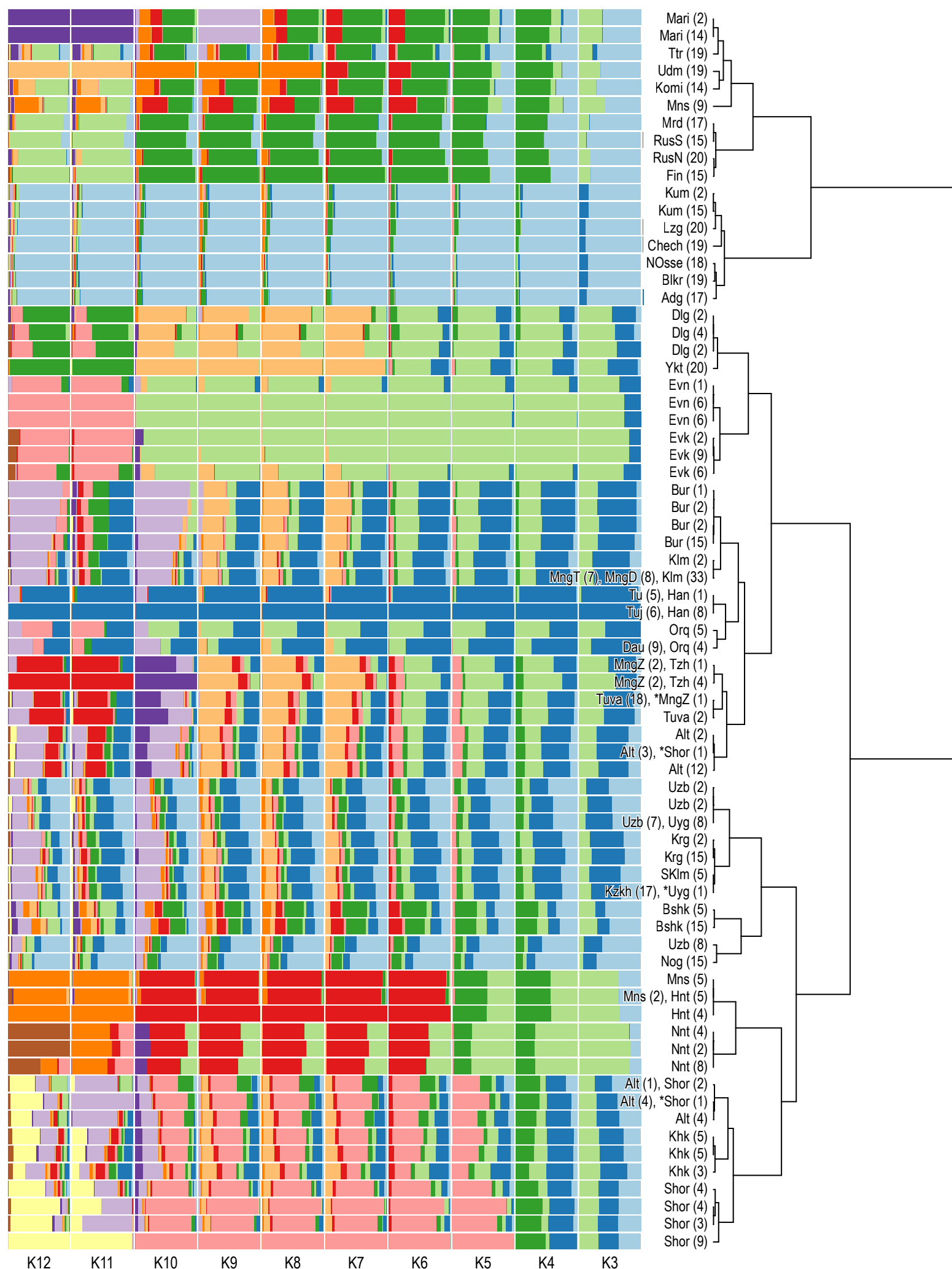

Supplement: Supplementary file 4 — Fig.S3 [file 41431_2024_1588_MOESM4_ESM.pdf]

# IBD sharing within each cluster

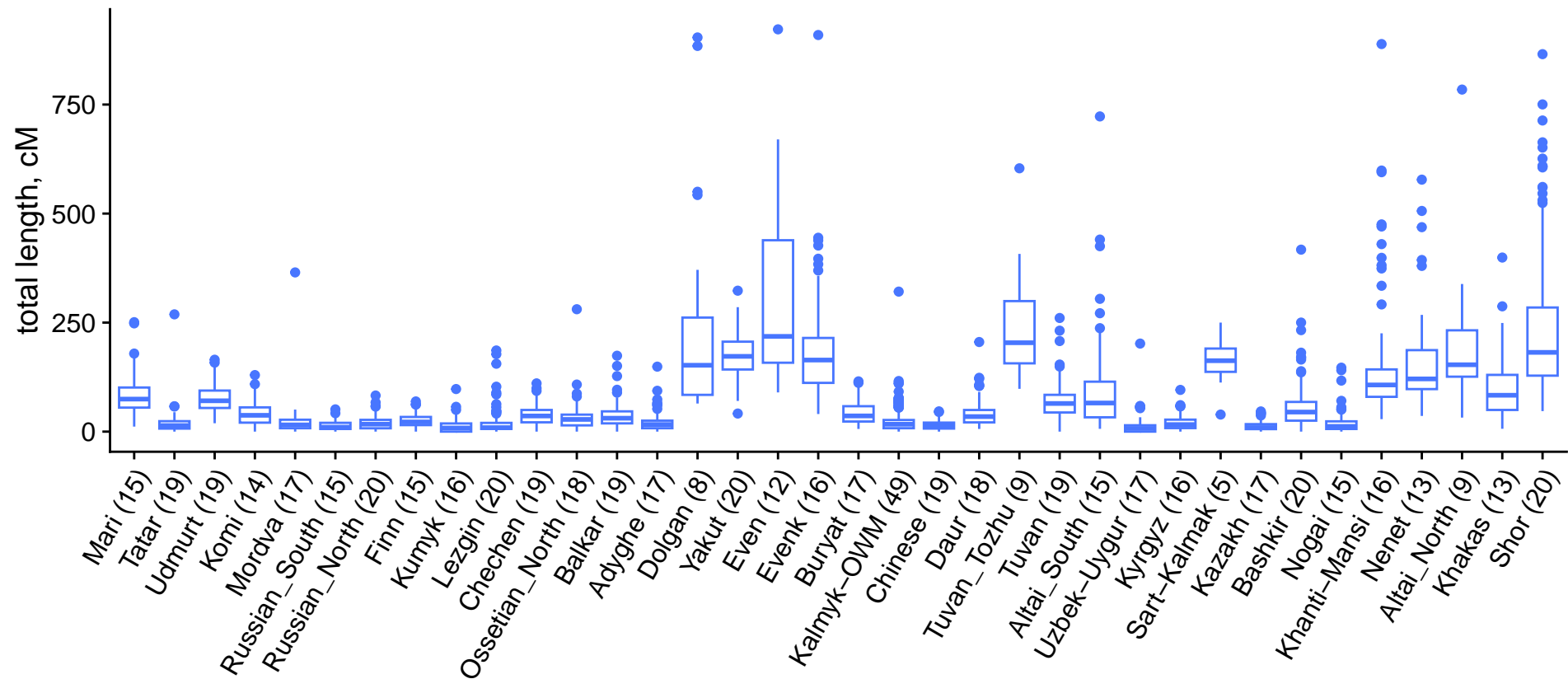

Supplement: Supplementary file 5 — Fig.S4 [file 41431_2024_1588_MOESM5_ESM.pdf]

# IBD sharing with each population

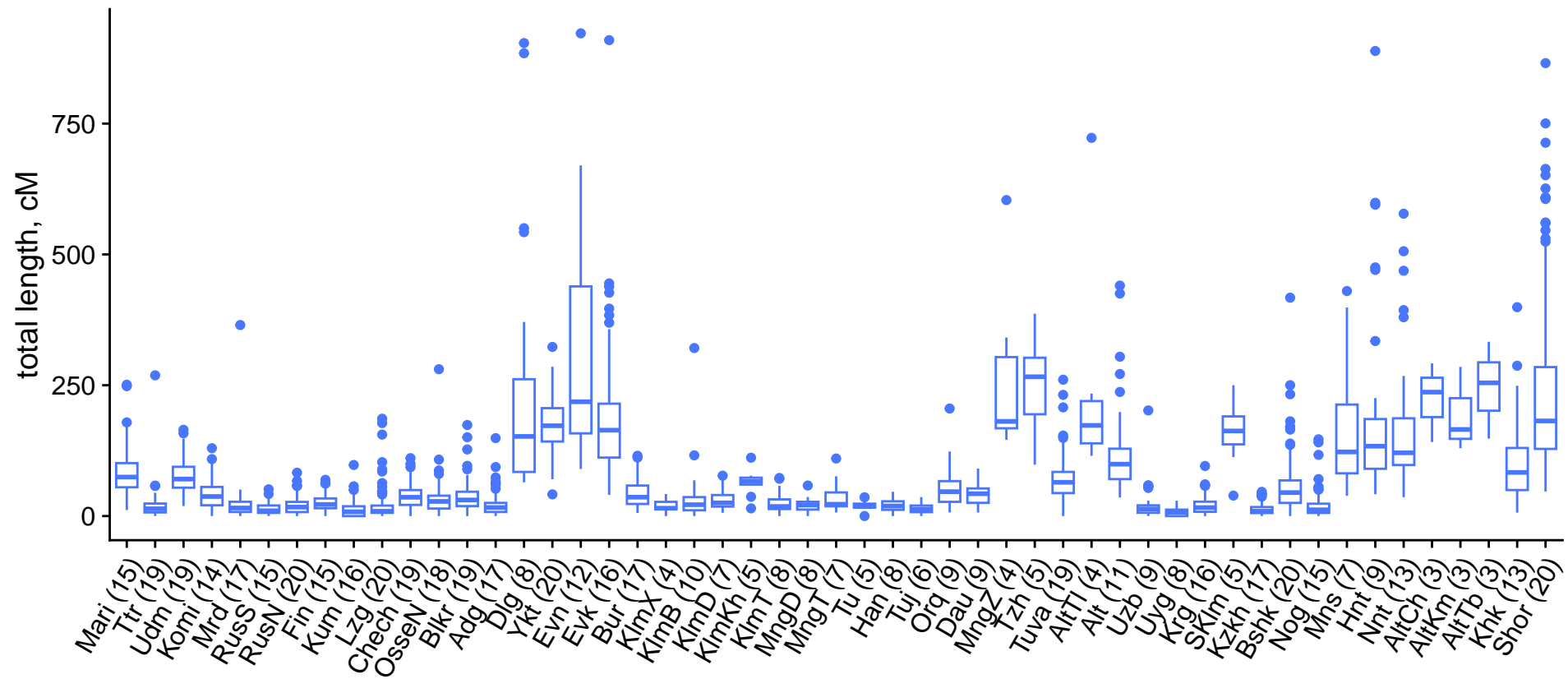

Supplement: Supplementary file 6 — Fig.S5 [file 41431_2024_1588_MOESM6_ESM.pdf]

Mean IBD total length, segments  $\geq 5$  cM

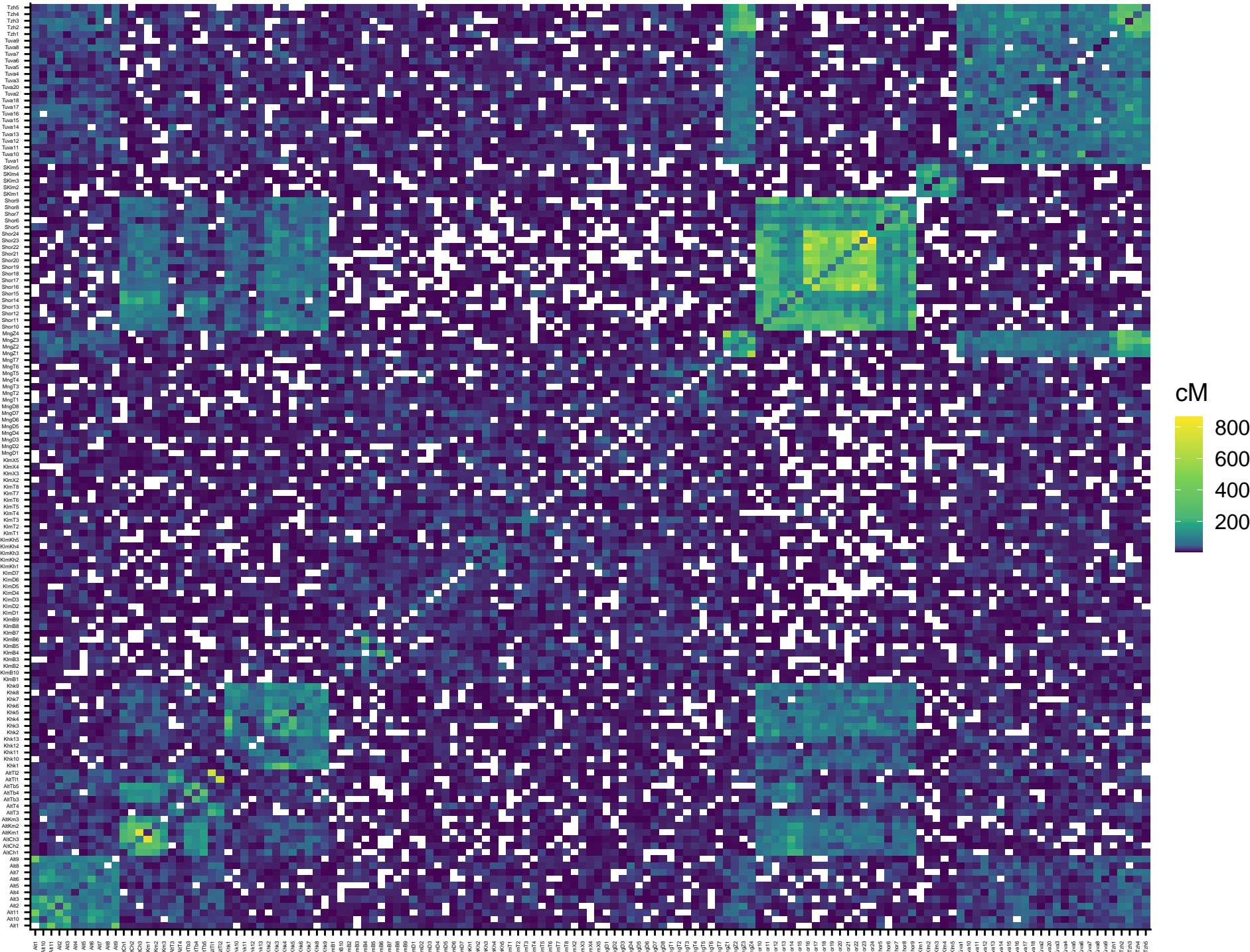

Supplement: Supplementary file 8 — Fig.S7 [file 41431_2024_1588_MOESM8_ESM.pdf]

# IBDNe for 50 Mongol and Kalmyk individuals

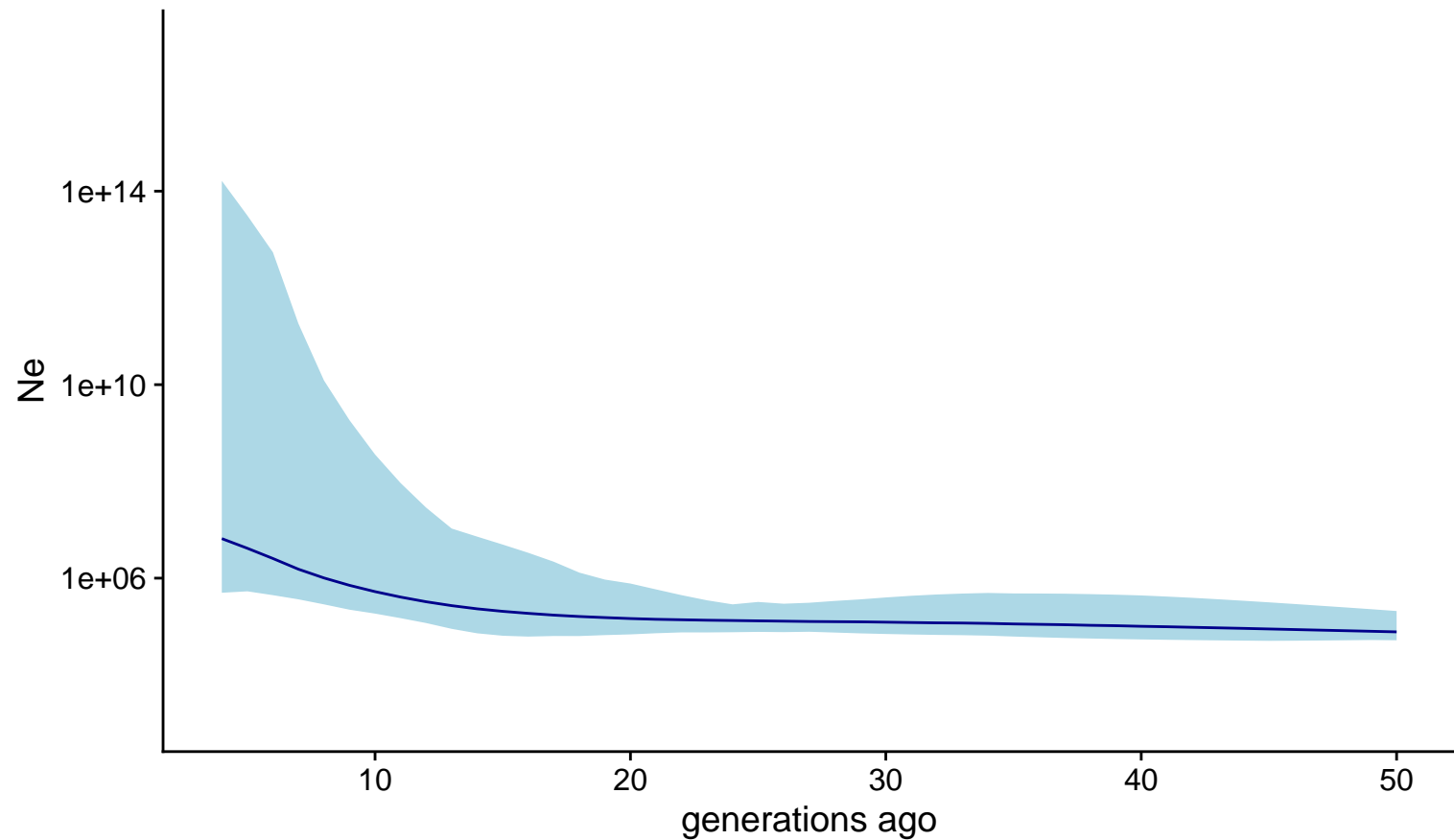

Supplement: Supplementary file 9 — Fig.S8 [file 41431_2024_1588_MOESM9_ESM.pdf]

A

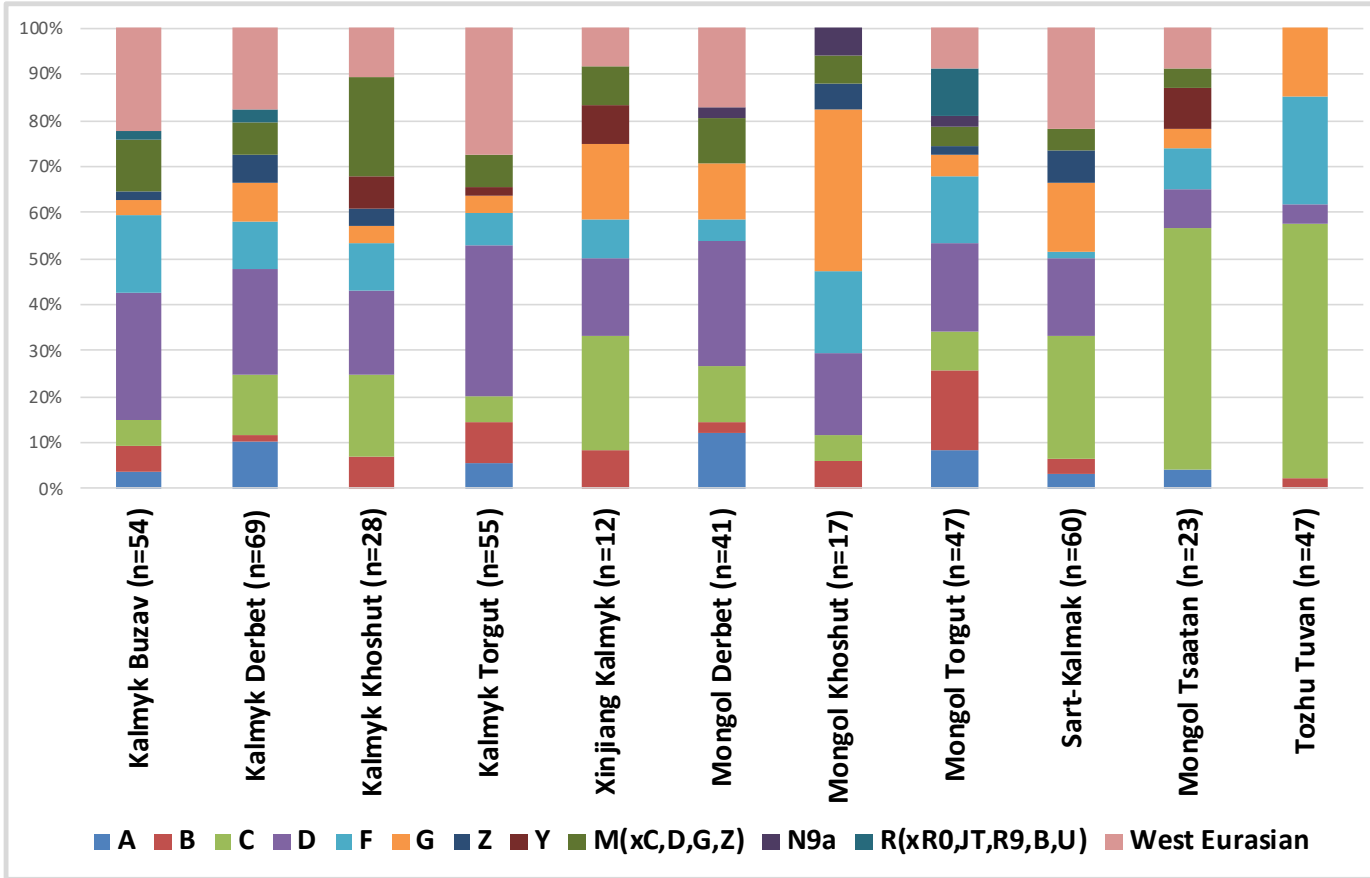

B

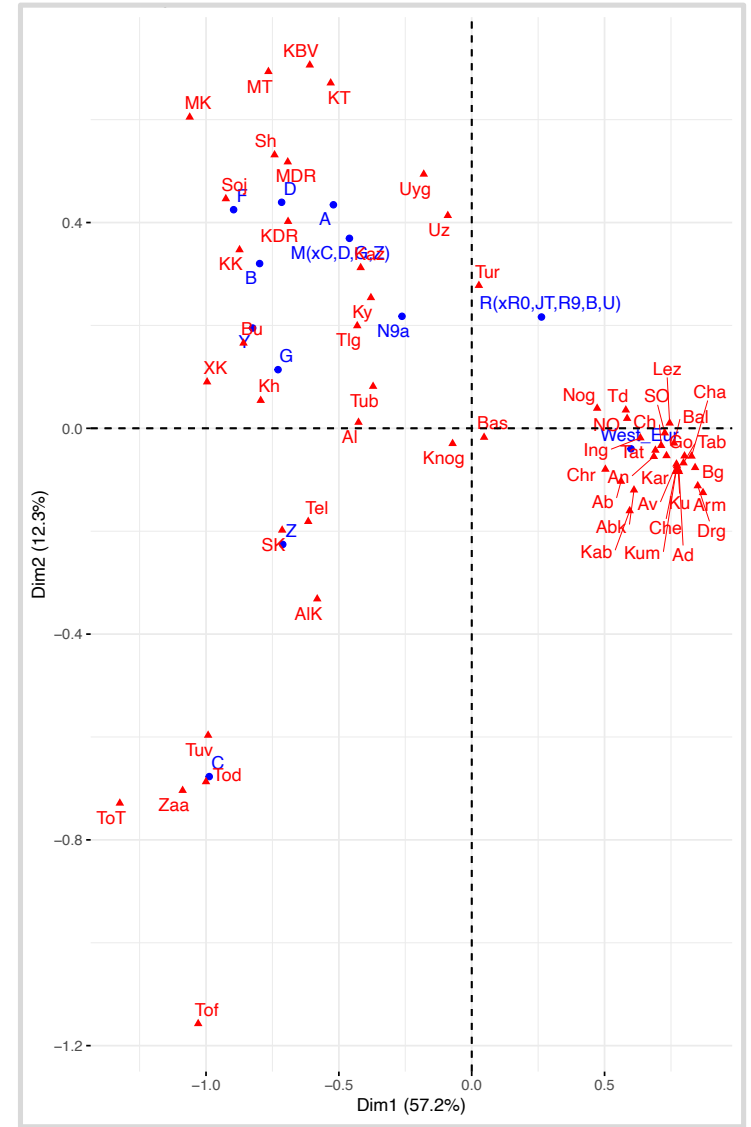

Supplement: Supplementary file 10 — Fig.S9 [file 41431_2024_1588_MOESM10_ESM.pdf]

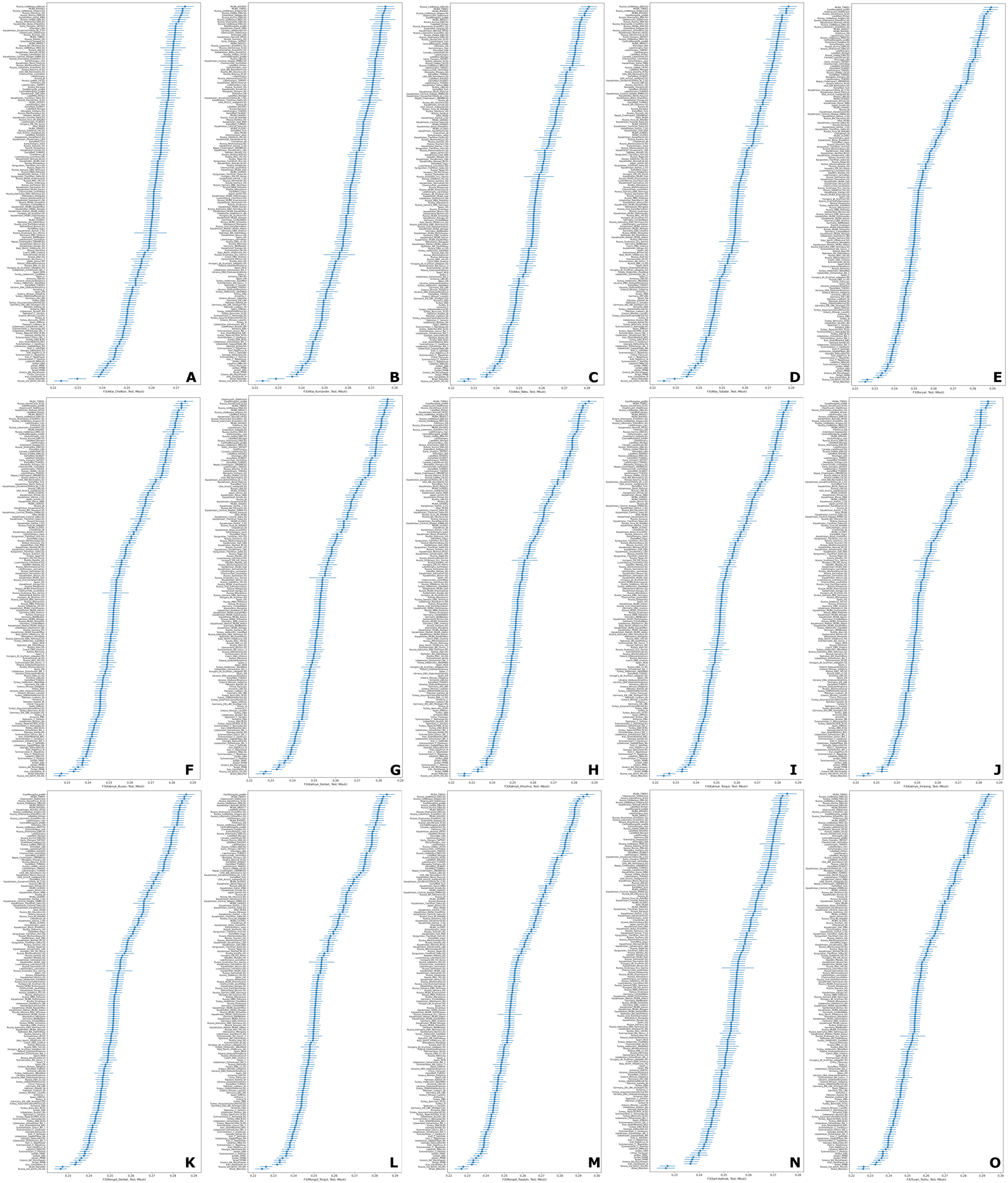

Supplement: Supplementary file 11 — Fig.S10 A-O [file 41431_2024_1588_MOESM11_ESM.pdf]
