# Supplementary material for: Gene pool preservation across time and space In Mongolian-speaking Oirats
Source: Eur J Hum Genet. 2024 Apr 11;32(9):1150–8. doi: 10.1038/s41431-024-01588-w (PMC11369229; doi:10.1038/s41431-024-01588-w)
Supplement: Supplementary file 7 — Fig.S6 [file 41431_2024_1588_MOESM7_ESM.pdf]

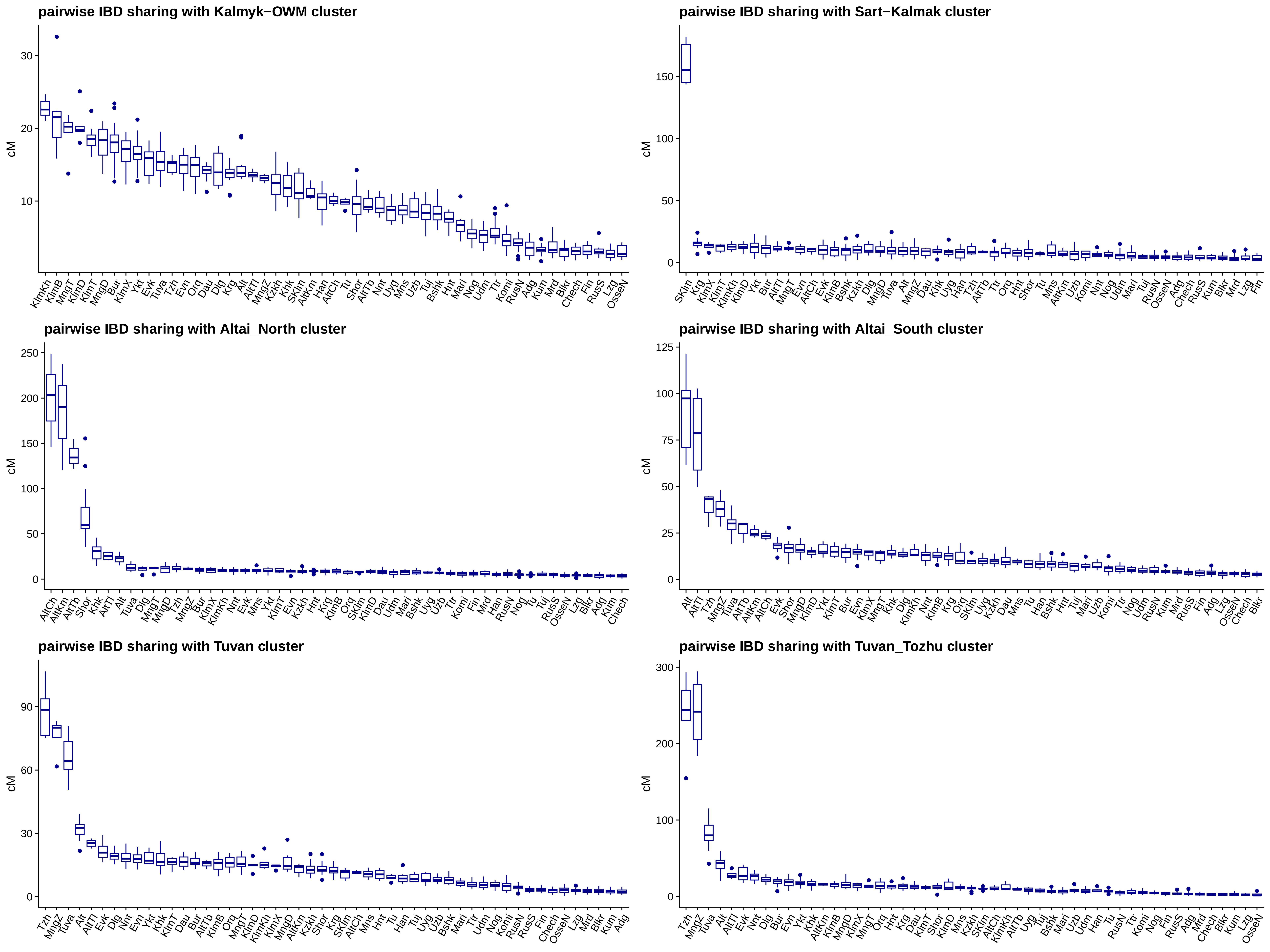
pairwise IBD sharing with Kalmyk−OWM cluster
cM
30
20
10
KlmKh KlmB MngT KlmD KlmT MngD Bur KlmX Ykt Evk Tuva Tzh Evn Orq Dau Dlg Krg Alt AltTl MngZ Kzkh Khk SKlm AltKm Han AltCh Tu Shor AltTb Nnt Uyg Mns Uzb Tuj Bshk Hnt Mari Nog Udm Ttr Komi RusN Adg Kum Mrd Blkr Chech Fin RusS Lzg OsseN
pairwise IBD sharing with Sart−Kalmak cluster
cM
150
100
50
0
SKlm Krg KlmX KlmT KlmKh KlmD Ykt Bur AltTl MngT Evn AltCh Evk KlmB Bshk Kzkh Dlg MngD Tuva Alt MngZ Dau Khk Uyg Han Tzh AltTb Ttr Orq Hnt Shor Tu Mns AltKm Uzb Komi Nnt Nog Udm Mari Tuj RusN OsseN Adg Chech RusS Kum Blkr Mrd Lzg Fin
pairwise IBD sharing with Altai_North cluster
cM
250
200
150
100
50
0
AltCh AltKm AltTb Shor Khk AltTl Alt Tuva Dlg MngT MngD Tzh MngZ Bur KlmX KlmKh Nnt Evk Mns Ykt KlmT Evn Kzkh Hnt Krg KlmB Orq SKlm KlmD Dau Udm Mari Bshk Uyg Uzb Ttr Komi Fin Mrd Han RusN Nog Tu Tuj RusS OsseN Lzg Blkr Adg Kum Chech
pairwise IBD sharing with Altai_South cluster
cM
125
100
75
50
25
0
Alt AltTl Tzh MngZ Tuva AltTb AltKm AltCh Evk Shor MngD KlmD Ykt KlmT Bur Evn KlmX MngT Khk Dlg KlmKh Nnt KlmB Krg Orq SKlm Uyg Kzkh Dau Mns Tu Han Bshk Hnt Tuj Mari Uzb Komi Ttr Nog Udm RusN Kum Mrd RusS Fin Adg Lzg OsseN Chech Blkr
pairwise IBD sharing with Tuvan cluster
cM
90
60
30
0
Tzh MngZ Tuva Alt AltTl Evk Dlg Nnt Evn Ykt Khk KlmT Dau Bur AltTb KlmB Orq MngT KlmD KlmKh KlmX MngD AltKm Kzkh Shor Krg SKlm AltCh Mns Hnt Tu Han Tuj Uyg Uzb Bshk Mari Ttr Udm Nog Komi RusN RusS Fin Chech OsseN Lzg Mrd Blkr Kum Adg
pairwise IBD sharing with Tuvan_Tozhu cluster
cM
300
200
100
0
Tzh MngZ Tuva Alt AltTl Evk Nnt Dlg Bur Evn Ykt Khk AltKm KlmB MngD KlmX MngT Hnt Krg Dau KlmT Shor KlmD Mns Kzkh SKlm AltCh KlmKh AltTb Uyg Tuj Bshk Mari Uzb Udm Han Tu RusN Ttr Komi Nog Fin RusS Adg Mrd Chech Blkr Kum Lzg OsseN
